# Properties of Stone Matrix Asphalt Modified with Polyvinyl Chloride and Nano Silica

**DOI:** 10.3390/polym13142358

**Published:** 2021-07-19

**Authors:** Hoang Phong Nguyen, Peifeng Cheng, Tat Thang Nguyen

**Affiliations:** 1 Civil Engineering Department, Northeast Forestry University, Harbin 150040, China; nhphongtdt@gmail.com; 2 College of Electromechanical and Civil Engineering, Vietnam National University of Forestry, Xuan Mai, Hanoi 13417, Vietnam; 3 College of Wood Industry and Interior Design, Vietnam National University of Forestry, Xuan Mai, Hanoi 13417, Vietnam; thangnt@vnuf.edu.vn

**Keywords:** polyvinyl chloride, nano silica, stone matrix asphalt, rolling thin film oven, asphalt binder

## Abstract

In this study, the effects of polyvinyl chloride (PVC) and nano silica (NS) as modifiers on the properties of stone matrix asphalt (SMA) were studied. The experiment was performed with five modes: 1% NS was mixed into SMA; 5% PVC was mixed into SMA; and the ratio of NS was changed (1, 2, and 3%) with 5% PVC being mixed into SMA. The properties of modified and unmodified SMA materials were determined and compared by performing the penetration test, softening points test, viscosity measurements, dynamic shear rheometry, and multiple stress creep recovery under aging conditions. Moreover, the properties of the modified SMA were also determined in terms of Marshall stability, water stability, and rutting resistance. The obtained results indicate that the physical properties of SMA materials could be significantly improved by using a combination of PVC and NS as a modifier. Moreover, the SMA mixtures modified with PVC and NS exhibited high Marshall stability, good moisture damage resistance, and rutting resistance. Modified SMA mixtures with 5% PVC and 1% NS exhibited the best quality. This research has opened up a new avenue for the development of effective additives for SMA materials.

## 1. Introduction

In recent years, the highway pavement construction industry has rapidly progressed at the global scale due to the dramatic increase in traffic loads. Many studies on improving the quality of road materials have been reported recently [1,2,3,4,5,6,7,8].

Stone matrix asphalt (SMA) is a type of asphalt mixture which is composed of asphalt, fiber stabilizer, mineral powder, fine aggregate, and coarse aggregate. It exhibits the characteristics of high content of coarse aggregate and mineral powder, large amounts of asphalt, and low content of intermediate particles. Asphalt is filled in the gap of the coarse aggregate skeleton, which results in an asphalt mixture with dense skeleton structure. The skeleton, which can bear heavy loads, results from the direct contact and tight interlock of the large number of aggregates. In contrast, the mastic with high bonding strength, which can improve the overall mechanical properties of the SMA, is formed through interaction between asphalt and mineral powder. The combination of skeleton and mastic endows the SMA with enough vertical and lateral restraint and the ability to resist permanent deformation under the vehicle load. Nowadays, SMA is widely used in asphalt road construction because of its good temperature stability, excellent skid resistance, durability, and rutting resistance [9].

Recently, it has been reported that the use of additives such as polymers and inorganic compounds in modifying asphalt materials is a way to generally improve the behavior of asphalt and SMA [10,11]. Different types of modifiers result in different characteristics of asphalt and SMA. Polyvinyl chloride (PVC) is a very popular plastic used extensively all over the world. However, large amounts of PVC are wasted every year, which also causes serious environmental pollution [12]. Thus, efficient use of waste PVC has become imperative for effective nationwide waste management performance. Over the years, waste PVC has been used as a modifier in asphalt and SMA modification [13,14,15]. Researchers have found that PVC addition in asphalt has a positive influence on the strength, stability, rutting resistance, rheological properties, and temperature sensitivity of the asphalt mixture [16]. Furthermore, the stiffness and Marshall stability of the SMA could also be enhanced, while the deformation under heavy traffic loads at high temperatures, the final strain, and the cost of pavement production could be decreased after adding PVC in asphalt [17,18].

In order to enhance the properties of asphalt, some recent studies have mixed with asphalt [12,19,20,21,22,23,24,25]. Nano silica (SiO_2_) (NS), which is an inorganic compound, is also widely used as an additive to improve the properties of the asphalt material. In recent years, NS has attracted significant research attention for modifying asphalt because of its high stability, high surface area, strong adsorption, and good dispensability [26,27,28]. Furthermore, NS has fascinating and outstanding properties in terms of strength, toughness, and thermal stability at high temperature [29,30,31]. Researchers have found that the addition of NS in asphalt could improve its strength, durability, and resistance against raveling and cracks and reduce its costs during its service life [32,33,34]. Therefore, PVC and NS act as promising candidates for asphalt modifiers.

Although the advantages of using PVC and NS, separately, in asphalt modification have already been reported in the literature, the combination of PVC and NS to enhance the quality of asphalt has not been studied to date. Therefore, the main objective of this study was to explore the synergistic effect of PVC and NS as modifiers on the properties of SMA and an attempt was made to develop a new additive for asphalt.

## 2. Materials and Methods

### 2.1. Materials

The asphalt in this study was AH-70, which was bought from Shanghai Wanzhao Co. Ltd. Shanghai, China. The basic properties of the asphalt are shown in Table 1.

The PVC powder used in this study was made from plastic waste. The basic properties of the PVC powder are shown in Table 2.

The NS powder was purchased from Nanjing Paukert Advanced Co., Ltd., Nanjing City, China. The basic properties of the NS powder are shown in Table 3.

Lignin fiber was purchased from Funa New Material Technology Co., Ltd., Xiamen City, China. The basic properties of the lignin fiber are shown in Table 4.

The aggregates were provided by Suke Environmental Protection Technology Co., Ltd., Suzhou City, China. The basic properties of the aggregates are shown in Table 5.

**Figure 1:**
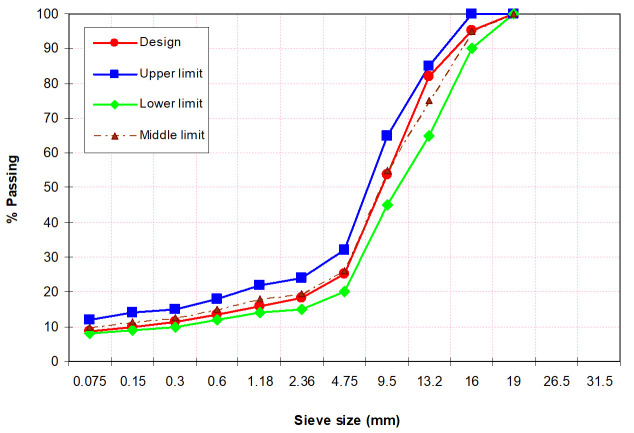
Gradation of aggregates.

The gradation limits of the aggregates used in this study are determined based on Chinese Standard JTG F40-2017 (SMA-16) and are shown in Figure 1.

### 2.2. Experimental Design

**Figure 2:**
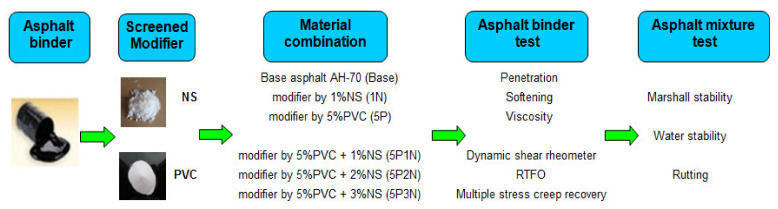
Experimental plan.

A flow chart is given to illustrate the experimental process in Figure 2.

### 2.3. Asphalt Modification

The asphalt was modified as follows. Original asphalt (about 500 g) was heated to 170 °C. Then, PVC (5%) was added gradually to the asphalt while being stirred at a constant speed of 4000 r/min for 60 min at 180 °C with a mechanical mixer. After that, NS (1%, 2%, 3%) was added while being stirred at a constant speed of 3500 r/min for 45 min at 160 °C. Finally, the mixture was stirred at a low speed of 200 r/min for 15 min to remove the air introduced during high-speed mixing.

### 2.4. SMA Formulation

The binder-aggregate and the fiber-aggregate ratios of SMA are shown in Table 6.

### 2.5. Asphalt Test

Penetration, softening point, rotational viscosity, dynamic shear rheometer (DSR), multiple stress creep recovery (MSCR), and standard rolling thin film oven (RTFO) experiments were conducted on the original and modified asphalt according to ASTM D5, ASTM D36, ASTM D4402, ASTM D7175-08, ASTM D7405, and ASTM D2872 (ASTM, 2015), respectively.

### 2.6. SMA Test

#### 2.6.1. Marshall Test

Specimens for the Marshall test are manufactured following the literature [35]. Four replicates of the specimens for each group (101.6 mm in diameter and 63.5 mm in height) were produced with 75 blows of compacting energy per side. Before measuring the MS, the specimens were placed in 60 °C hot water for 30 min for maintenance.

#### 2.6.2. Moisture Susceptibility Test

The moisture susceptibility of the SMA was determined through indirect tensile strength (ITS) tests according to the AASHTO T283. The ITS and tensile strength ratio (TSR) values were measured using the following Equations (1) and (2) [36]. The moisture susceptibility tests were repeated 6 times.

Indirect tensile strength (ITS):

(1) $$\mathrm{ITS}=\frac{2.P_{max}}{\pi .h.d}\times 100$$

ITS: indirect tensile strength (MPa);

P_max_: peak load (N);

h: height of sample = 63.5 (mm);

d: diameter of sample = 101.6 (mm).

Tensile strength ratio (TSR):

(2) $$\mathrm{TSR}=\frac{ITS_{wet}}{ITS_{dry}}\times 100$$

TSR: Tensile strength ratio (%);

ITS_wet_ is the indirect tensile strength at wet condition (MPa);

ITS_dry_ is the indirect tensile strength at dry condition (MPa).

#### 2.6.3. Rutting Test

The rutting was conducted according to the JTG E20-2011 standard. The test samples were prepared by the wheel grinding method [37]. The length, width, and thickness of the sample were 300 mm, 300 mm, and 50 mm, respectively. The temperature was 60 °C, the load was 0.7 MPa, and the rolling rate was 42 times/min. The dynamic stability (DS) was measured as follows:

(3) $$\mathrm{DS}=\frac{42\times 15}{d_{60}-d_{45}}$$

DS: dynamic stability (cycles/mm);

d_60_, d_45_: rut depth (mm) at 60 min and 45 min, respectively;

42: wheel speed per min (cycles/min);

15: the time difference (min).

## 3. Results and Discussion

### 3.1. Penetration

**Figure 3:**
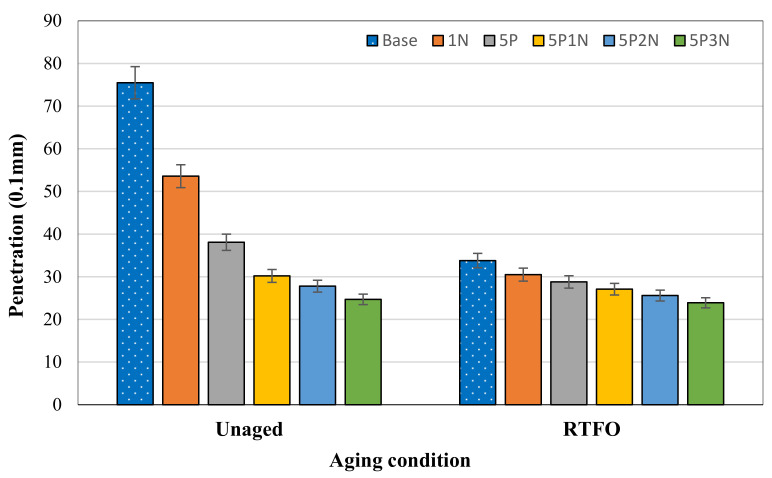
Penetration of AH-70 asphalt modified with PVC and NS.

Penetration is the consistency of the material and reflects the rheological properties of the asphalt binder. Figure 3 indicates the effect of PVC, NS, and PN on the penetration of the modified asphalt under short-term aging. Adding PVC, NS, and PN made the penetration value of virgin asphalt decrease significantly. The measured penetration depth of asphalt decreased by 29% at a 1% NS content, by 49.53% at a 5% PVC content, and by 60% at the 5% PVC + 1% NS content. The penetration depth of the asphalt binder modified with PN was lower than those of PVC- and NS-modified binders; the lowest penetration depth value was at the 5P3N. Furthermore, asphalt samples modified with PN represented the continuous penetration decrease for unaged and RTFO-aged asphalt. The anti-aging property of asphalt was strengthened after the addition of PVC and NS.

### 3.2. Softening Point

**Figure 4:**
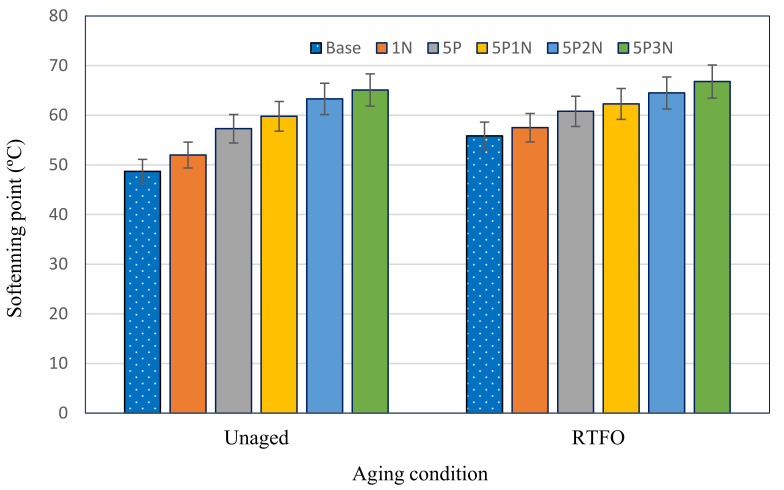
Softening point of AH-70 asphalt modified with PVC and NS.

The softening point describes the high temperature stability of the binder in the laboratory. Normally, a high softening point implies that the asphalt possesses high temperature stability. Figure 4 shows the effects of PVC and NS with various concentrations on the softening points of unmodified and modified asphalt before and after short-term aging. The softening point of base asphalt climbed from 48.7 °C to 55.85 °C after RTFO aging. Additionally, the softening point rose with the rise in NS content; even though the increment was negligible at all concentrations, the highest value was at 5P3N. The combination of PVC and NS additives increased the softening point value, indicating that adding PN improved the stability of asphalt under high temperature.

### 3.3. Rotational Viscosity

**Figure 5:**
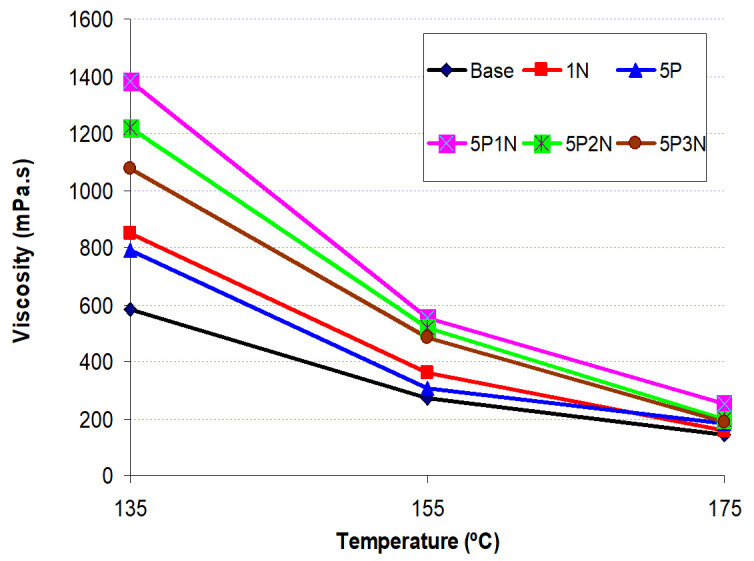
Viscosity of base asphalt, and five types of modified asphalt with different temperature.

The effect of rotational viscosity on the asphalt binder’s workability is very important in selecting proper mixing and compacting temperature [38]. The rotational viscosity reflects the flow property of the asphalt binder. Figure 5 shows the rotational viscosity of base MSA and five types of modified asphalt in the temperature range from 135 °C to 175 °C.

Figure 5 shows that the rotary viscosity value of five types of modified asphalt is higher than base MSA (the rotational viscosity value from 800 to 1400 mPa.s). These results show that, when adding PVC, NS or a mixture of PVC and NS to MSA, the rotational viscosity of the MSA mixture will increase, which is vital in increasing the binder film thickness for coating aggregates in the hot mixture. Therefore, the high viscous MSA mixture will maintain the stability of asphalt mixtures [39]. The rotational viscosity values became highest at the ratio 5P1N.

### 3.4. Dynamic Shear Rheological Properties

**Figure 6:**
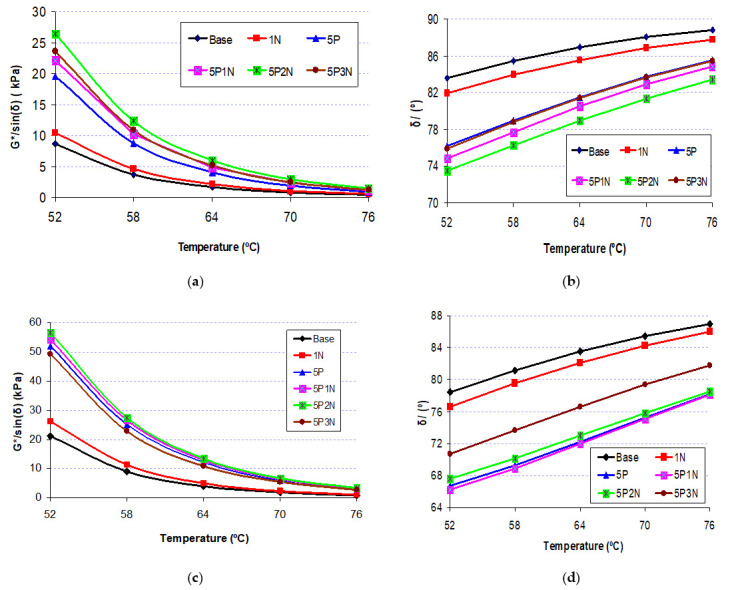
G*/sin(δ) and (δ) of unaged and aged binder with PVC and NS contents. (**a**) Unaged; (**b**) Unaged; (**c**) RTFO-aged; (**d**) RTFO-aged.

The DSR experiment was performed to test the rutting resistance of the asphalt binder under high temperatures by measuring G*/sin(δ). The asphalt binder needs to be sufficiently flexible to prevent rutting. The value of G*/sin(δ) illustrates the performance grade of asphalt. Figure 6 represent the rutting parameters of PVC, NS and PN measured at temperatures of 52 °C, 58 °C, 64 °C, 70 °C and 76 °C before and after RTFO aging. The results show that G*/sin(δ) increases as the concentration of modifiers increases. Moreover, the G*/sin(δ) values of the PVC- and PN-modified asphalt were higher than for the NS-modified asphalt. This means that the asphalt binder modified with PVC and PN had higher anti-rutting properties than NS counterparts. In particular, binder 5P2N shows the highest rutting factor values, three times and two times higher as compared to the base asphalt before and after RTFO aging, respectively. Therefore, the asphalt binder modified with PN can provide the best performance at higher temperatures.

The change in phase angle (δ) can be used to assess the anti-aging of RTFO-aged modified asphalt. A decrease in (δ) enhances the elastic reply of the asphalt and mixture flexibility. As shown in Figure 6c, the (δ) of all RTFO-aged binders are much lower than that of their virgin asphalt. On the other hand, in Figure 6d, the (δ) value of series “Base” increased from 78° to 87° and also increased from 67° to 78° in series “5P”. In the meantime, series “5P2N” has the lowest (δ) value among all types of asphalt binder mixtures. This indicates that the modified asphalt possesses a more elastic structure after RTFO aging and could enhance the asphalt binder’s anti-rutting property.

### 3.5. Multiple Stress Creep Recovery (MSCR)

**Figure 7:**
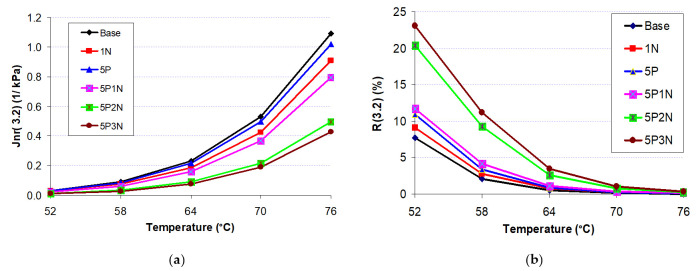
MSCR parameters at different temperature: (**a**) average non-recoverable creep compliance (J_nr_) at 3.2 kPa; (**b**) average percent recovery (R) at 3.2 kPa.

J_nr_(3.2) predominantly characterizes the anti-rutting of an asphalt binder, and typically, a higher J_nr_(3.2) value represents a lower rutting performance. Figure 7a shows that the J_nr_ values for all the asphalts decreased with PVC, NS, and PN additives with the increase in temperature. With respect to rutting resistance, the 5P3N binder illustrated the best performance, and the base asphalt ranked the lowest.

The measure R(3.2) was used for evaluating the delayed elastic conduct of the asphalt binder. A larger R can be interpreted as a larger capacity of bitumen to recover from deformation tested with the applied load. In general, 5P3N asphalt had the best performance under most cases, with varied testing temperature as well as varied PVC and NS contents. This means that 5P3N asphalt has the greatest elastic structures when subjected to creep-recovery loading alignments Figure 7b. The virgin asphalt exhibited the lowest recovery properties at all temperatures.

This result suggests that the addition of PVC and NS improves the elastic response of the virgin binder, enhancing the capability for anti-rutting under repeated loads of asphalt binder.

### 3.6. Short-Term Aging Effect

The rolling thin-film oven (RTFO) test is one of the most commonly used standardized tests to simulate the short-term aging (STA) of binders. This test is used to measure the combined effects of heat and air on a thin film of asphalt or bituminous binder in permanent renewal. It aims to simulate the hardening that a bituminous binder undergoes during the mixing, transporting, and compacting processes, referred to as STA. The aging indices ratio (AIR) values measured for the asphalt binder modified with NS and PVC before and after aging at different temperatures are shown in Table 7. From Table 7, it can be seen that when the temperature changes from 52 °C to 76 °C, the AIR values of the material also change. However, the AIR values of the material mixtures did not change too much. It can be seen that when given NS or PVC individually, the AIR values are higher than those of base asphalt. When combining NS and PVC into the asphalt mixture, the AIR value decreases gradually. With 5P2N and 5P3N compounds, the AIR values are lower than those of base asphalt.

### 3.7. Marshall Experiment

**Figure 8:**
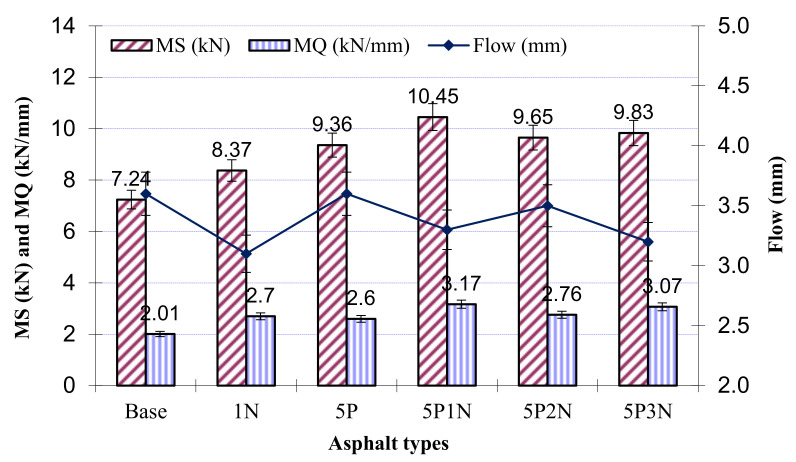
Results of MS experiment.

Four specimens for the modified and unmodified SMA mixtures were manufactured at the optimum asphalt-aggregate ratio (Table 6) and used to measure the characters of the SMA mixture specimens by the Marshall experiment. It is clear that the use of asphalt with PVC and NS meets the significant requirements to be applied over roads. MS results from Figure 8 show that the addition of only PVC, only NS or both PVC and NS in all situations does not reach the lowest value (i.e., base value). Furthermore, regarding the SMA mixture with virgin asphalt, MS increased by 15.6% for asphalt concrete with 1% NS, 29.28% for asphalt concrete with 5% PVC, 33.28% with 5P2N, 35.77% with 5P3N, and most notably, 44.33% with 5P1N. The Marshall Quotient (MQ) was measured by MS/flow and it was clearly obvious that the MQ value increased significantly with the addition of PVC and NS. This conduct could be explained by the fact that PVC and NS fragments operate as a stiffener in the SMA concrete, filling voids between rough aggregates, and also operating as a bridge between the aggregates and the asphalt cement binder. The addition of NS increases the mixture stability while decreasing the flow value, which indicates that NS enhanced the deformability resistance of the SMA mixture. This indicates that SMA mixtures modified with PVC and NS may be useful for pavement areas where high stability and stiffness are needed.

### 3.8. Moisture Susceptibility Test

**Figure 9:**
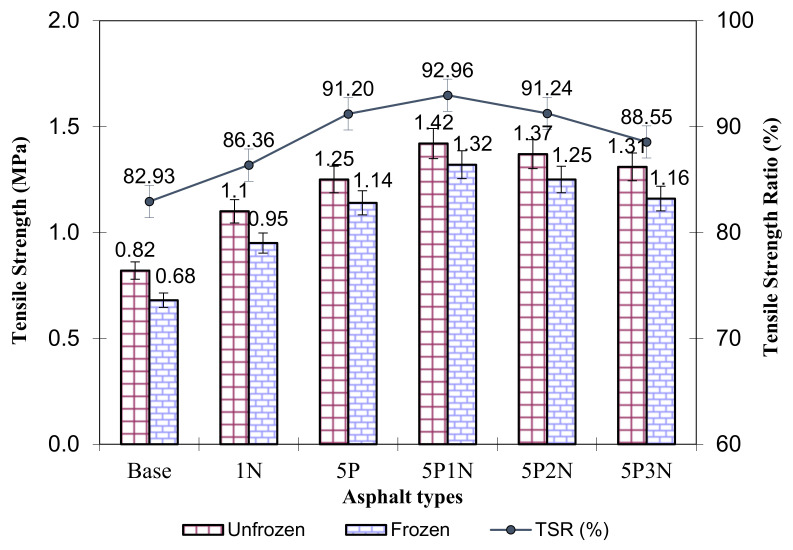
Results of moisture susceptibility experiment.

Figure 9 displays the changes in the ITS and TSR values when PVC and NS are added. The ITS value of the virgin SMA mixture modified with PVC and NS was higher than in the unmodified SMA mixture. The experimental results displayed that the TSR values of the base asphalt specimen and the highest specimen modified with 5P1N were 82.93% and 92.96%, respectively. NS possesses very small fragment sizes and large specific surface area so that it can absorb more free binders and increase the structural binder. Working with adding PVC and NS, the moisture sensitivity of the composed modified SMA is reduced. It has also been shown that 5P1N-modified asphalt mixture has the highest tensile strength value (Figure 9).

The ITS and TSR values rose when modifiers were added at standard testing temperatures, due to an increased grade of adhesion between the aggregates and binder, leading to a decrease in the grade of stripping of the SMA mixture. Some previous studies have shown that, when adding plastic polymer to asphalt, the ITS and TSR values increase [40,41]. When adding NS to the asphalt, the ITS and TSR values also increased [42,43]. Therefore, this suggests that the addition of PVC and NS leads to an increase in the ITS and TSR values; thus, the moisture susceptibility of the SMA mixture is also enhanced.

### 3.9. Rutting Test

**Figure 10:**
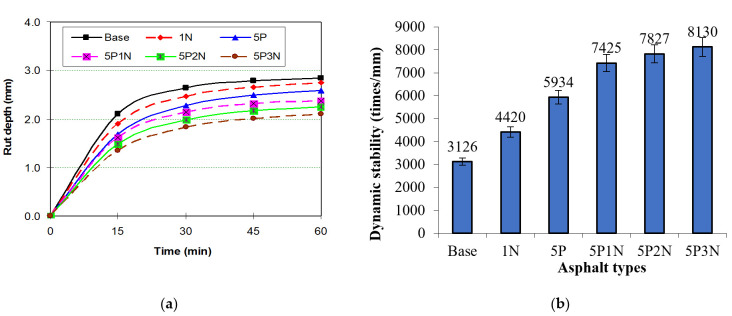
Relationship between PVC and NS content and rutting properties ((**a**)—rut depth, (**b**)—dynamic stability).

The results of the rutting test are shown in Figure 10. Adding PVC and NS led to a decrease in rut depth. Using Equation (3), the dynamic stability has been determined and described (Figure 10). Figure 10b exhibits that the DS of the base asphalt mixture was 3126 cycles/mm, while that for the SMA mixture modified with 1% NS was 4420 cycles/mm; 5% PVC was 5934 cycles/mm, and 5P3N was 8130 cycles/mm. Moreover, the DS value increased by 40.94% with 1% NS, by 89.22% with 5% PVC, and by 160% with 5P3N. The addition of PVC and NS is the reason why SMA mixture strength and stiffening were improved. This shows a climb in the capability of the SMA mixture to resist accumulating deformations under heavy traffic loads. Thus, by adding PVC and NS, the deformation resistance of the SMA mixture is improved.

## 4. Conclusions

In this study, the properties of stone matrix asphalt (SMA) were enhanced by adding polyvinyl chloride (PVC) and nano silica (NS) as a combined modifier. Based on the results, the following conclusions can be drawn:

When PVC and NS were added separately to the SMA materials, the physical and mechanical properties of the SMA mixtures were improved. When both NS and PVC were added together into the SMA mixtures, the properties of the material improved significantly. The modified SMA mixtures exhibited enhanced resistance to deformation under high temperatures; increased resistance to subsidence; improved water resistance; and good anti-aging ability.

SMA mixtures with 5% PVC and 1% NS exhibited the best quality. The research has opened up a new avenue for the development of effective additives for SMA materials.

## Figures and Tables

**Table 1:** Basic properties of the asphalt.

Basic Properties	Method	Value	Unit
Penetration (100 g, 5 s, 25 °C)	ASTM D5	75.5	0.1 mm
Ductility (25 °C cm/min, 5 cm/min)	ASTM D113	>150	cm
Softening point	ASTM D36	48.7	°C
Rotation viscosity (135 °C)	ASTM D4402	0.581	Pa. s
Specific gravity at 25 °C	ASTM D70	1.04	g/cm^3^
Flash point	ASTM D92	320	°C

**Table 2:** Properties of PVC powder.

Appearance	Melting Point (°C)	Density (g/cm^3^)	Tensile Strength (MPa)
White	170	1.42	60

**Table 3:** Properties of NS powder.

Type	Appearance	Silica Content (%)	Average Grain Size (nm)	Specific Surface Area (m^2^/g)	pH Value
SP15	White	99.8	15 ± 5	250 ± 30	5–7

**Table 4:** Properties of lignin fiber.

Appearance	Length (mm)	Diameter (μm)	Density (g/m^3^)	pH Value
Gray	<5	46	1.6	7.5 ± 1

**Table 5:** Properties of aggregates.

Aggregate Properties	Method	Value	Unit
Crushed stone value	ASTM D6928	13.1	%
Los Angeles abrasion loss	ASTM C131	15.9	%
Apparent specific gravity	ASTM C128	2.78	g/cm^3^
Water absorption	ASTM C70	0.41	%
Fine aggregate specific gravity	ASTM C127	2.78	g/cm^3^
Sand equivalent	ASTM D2419	75.6	%

**Table 6:** Binder-aggregate and fiber-aggregate ratios of mixtures.

Binder Type	Binder-Aggregate Ratio (%)	Fiber-Aggregate Ratio (%)
Control	6.1	0.28
1% NS	6.1	0.31
5% PVC	6.1	0.32
5P1N	6.2	0.33
5P2N	6.2	0.33
5P3N	6.2	0.33

**Table 7:** AIR values approximated for various types of asphalt at different temperatures.

Asphalt Type	Aging Indices at Difference Temperatures				
	52 °C	58 °C	64 °C	70 °C	76 °C
Base	2.44	2.47	2.38	2.28	2.15
1N	2.53	2.48	2.35	2.20	2.10
5P	2.66	2.88	3.04	3.10	3.11
5P1N	2.46	2.60	2.70	2.75	2.74
5P2N	2.15	2.23	2.29	2.32	2.30
5P3N	2.08	2.12	2.14	2.16	2.16

## Data Availability

The data presented in this study are available in this study. Additional information could be available on request from the corresponding author.
